# Supplementary material for: A multi-time scale rolling optimization framework for low-carbon operation of CCHP microgrids with demand response integration
Source: PLoS One. 2025 Jul 28;20(7):e0327523. doi: 10.1371/journal.pone.0327523 (PMC12303334; doi:10.1371/journal.pone.0327523)
Supplement: Attachment — Submitted filename: Response to Reviewers.docx [file pone.0327523.s002.docx]

**Responses to the reviewers' comments**

Dear editor,

Thank you for your letter and the reviewers’ comments on our manuscript entitled “A multi-time scale rolling optimization framework for low-carbon operation of CCHP microgrids with demand response integration”. We would like to thank the anonymous referees for their kind comments and valuable suggestions. We have studied the comments carefully and made corrections point-by-point. Revised portions are marked in red in the manuscript or in the supplementary material. The main corrections in the manuscript and the responses to reviewers’ comments are as follows.

Journal requirements:

Thank you for taking time out of your busy schedule to read our research work again. Your valuable suggestions are very helpful for the revision of our paper. We have read your opinions carefully and revised our manuscript. We hope this revision can meet your standards. Here are the point-to-point responses.

1. Please ensure that your manuscript meets PLOS ONE's style requirements, including those for file naming.

**Response**: We have revised the manuscript as PLOS ONE's style requirements, including those for file naming.

2. We note that the grant information you provided in the ‘Funding Information’ and ‘Financial Disclosure’ sections do not match.

**Response**: We provided the correct grant numbers for our study in the ‘Funding Information’ section.

3. Thank you for stating the following in the Acknowledgments Section of your manuscript: “This work was supported by the National Natural Science Foundation of China under Grant: 62333010.” We note that you have provided funding information that is currently declared in your Funding Statement. However, funding information should not appear in the Acknowledgments section or other areas of your manuscript. We will only publish funding information present in the Funding Statement section of the online submission form.

**Response**: The funding acknowledgments were omitted from the main text and instead provided through the online submission system.

4. When completing the data availability statement of the submission form, you indicated that you will make your data available on acceptance.

**Response**: We revised our statement. The original data were collected from an industrial park in Taizhou, Jiangsu Province, and provided by Company A under a non-disclosure agreement. To protect proprietary business information and comply with contractual obligations, the dataset has been anonymized and processed to remove sensitive details while retaining analytical utility.

5. PLOS requires an ORCID iD for the corresponding author in Editorial Manager on papers submitted after December 6th, 2016. Please ensure that you have an ORCID iD and that it is validated in Editorial Manager.

**Response**: We have an ORCID iD.

6. We note that Figure 1 in your submission contain copyrighted images.

**Response**: We update the Fig 1.

**Reviewer #1:**

Thank you for taking time out of your busy schedule to read our research work again. Your valuable suggestions are very helpful for the revision of our paper. We have read your opinions carefully and revised our manuscript. We hope this revision can meet your standards. Here are the point-to-point responses.

1. Since intra-day forecasting is introduced, short-term rolling optimization may suffer from myopic behavior compared to global optimization, which could negatively impact the overall cost. Can your method address this issue? Furthermore, given the real-time adjustment of schedules, the absence of anticipatory constraints might result in infeasibility in later stages. Would it be necessary to introduce slack variables to ensure feasible dispatch across stages?

**Response**: We sincerely appreciate your insightful feedback. Your observation regarding the potential myopic behavior of short-term rolling optimization and the risk of infeasibility due to real-time adjustments is indeed critical. Upon reflection, we recognize that our may not have fully addressed these challenges, and we appreciate the opportunity to clarify our methodology. We have modified the manuscript and added the following sentence to last paragraph of 4.1 section:

“This study presents a scenario-based Model Predictive Control (MPC) framework to address uncertainty in renewable energy systems while ensuring alignment between Real-time operational, Intra-day and Day-ahead optimization objectives. A double-layer forecasting approach is adopted for intra-day predictions, effectively mitigating the cost inefficiencies caused by myopic decision-making. Additionally, energy storage systems are leveraged to further compensate for forecasting errors and enhance operational flexibility. In the intra-day adjustment layer, MT_2_ is designated as the slack variable to absorb power imbalances, whereas in the real-time control layer, FC serves as the slack variable to ensure instantaneous power balance.”

2. The solution method for the proposed model is not clearly presented. I recommend elaborating on the algorithmic aspects and analyzing the scalability of the approach—namely, to what extent can your method handle large-scale problems while still meeting the real-time requirements?

**Response**: Thanks for your good suggestion. We have added the solution method for the proposed model. We also have modified the manuscript and added the following sentence to paragraph 2 of the 4.1 section:

“Model Predictive Control (MPC) is an advanced online control algorithm that optimizes system performance by predicting future outputs and states within a finite time horizon. This algorithm generates an optimal control sequence by continuously solving a constrained optimization problem based on real-time system states and environmental variations, thereby demonstrating superior control performance. For a multi-device system comprising N components, the predicted output sequence group at time step k for future j time steps can be mathematically expressed as follows:

$$y_{i}\left( k \right)=\left[ y_{i}\left( k+1\left| k \right. \right),y_{i}\left( k+2\left| k \right. \right),\ldots,y_{i}\left( k+j\left| k \right. \right) \right]$$

The sequence of corresponding control variables is as follow:

$$\Delta u_{i}\left( k \right)=\left[ u_{i}\left( k+1 \right)-u_{i}\left( k \right),\ldots,{u_{i}\left( k+j \right)-u}_{i}\left( k+j-1 \right) \right]=\left[ \Delta u_{i}\left( k \right),\ldots,\Delta u_{i}\left( k+j-1 \right) \right]$$

The system state variable sequence can be expressed as $x_{i}\left( k \right)=\left[ y_{i}\left( k \right),u_{i}\left( k \right) \right]$. The system constraints to be satisfied are as follows:

$$\left\{ \begin{aligned} \Delta u_{i,min}\left( k+j \right)\leq\Delta u_{i}\left( k+j \right)\leq\Delta u_{i,max}\left( k+j \right) \\ \Delta u_{i,min}\left( k+j \right)\leq\Delta u_{i}\left( k+j+1 \right)-\Delta u_{i}\left( k+j \right)\leq\Delta u_{i,max}\left( k+j \right) \\ y_{i,min}\left( k+j \right)\leq y_{i}\left( k+j \right)\leq y_{i,max}\left( k+j \right) \end{aligned} \right.$$

Where: $u_{i}\left( k+j \right)$ and $y_{i}\left( k+j \right)$ are the control and output variables of the i-th device at time $k+j$, respectively; $u_{i,max}\left( k+j \right)$, $u_{i,min}\left( k+j \right)$ , and $y_{i,max}\left( k+j \right)$ , $y_{i,min}\left( k+j \right)$ are the upper and lower constraints on the control and output variables at time $k+j$, respectively; $\Delta u_{i,max}\left( k+j \right)$ and $\Delta u_{i,min}\left( k+j \right)$ are the upper and lower constraints on the incremental amount of the control at time $k+j$, respectively.

The overall control strategy of the system can be expressed as follows:

$$F=min\sum_{m=1}^{j} P\left( y\left( k+m\left| k \right. \right)-y_{r}\left( k+m\left| k \right. \right) \right)^{2}+Q\left( u\left( k+m \right)-u\left( k+m-1 \right) \right)^{2}$$

Where: $y_{r}\left( k+m\left| k \right. \right)$ is the reference trajectory of the system at $k+m$ moments; $y\left( k+m\left| k \right. \right)$ and $u\left( k+m-1 \right)$ are the predicted output variables and control variables of the system at $k+m$ time, respectively; P and Q are the weight coefficient matrices of the output variables and control variables, respectively. In this study, the system outputs are defined as the electrical power purchased from the grid and the natural gas consumption. The control variables consist of the power outputs from the microturbine (MT), fuel cell (FC), energy storage (ES), electric boiler (EB), gas boiler (GB), and the integrated active power control signal (IASC). The predicted load variations, along with the forecasted power generation fluctuations from wind turbines and photovoltaic systems, are treated as system disturbances. Based on these definitions, a multi-input multi-output (MIMO) state-space model is established for the energy management system.”

3. Does the proposed mechanism support distributed implementation?

**Response**: The proposed mechanism in this study is inherently centralized and does not accommodate a distributed implementation framework. The Model Predictive Control (MPC) approach adopted here is specifically tailored for centralized scheduling applications, where computational efficiency and deterministic optimization are prioritized. In contrast, distributed scheduling systems entail higher-dimensional optimization problems, leading to substantially increased computational complexity and coordination challenges. To address these limitations, future research should investigate enhanced MPC algorithms with decentralized capabilities or hybrid methodologies that integrate Deep Reinforcement Learning (DRL) for improved scalability and adaptability. The key contribution of this work lies in its hierarchical optimization framework for intra-day scheduling, coupled with seamless integration into real-time optimization strategies, thereby enhancing operational efficiency and decision-making precision.

4. How accurate is the proposed forecasting model? A comparative analysis with other existing forecasting methods is suggested to highlight its effectiveness.

**Response**: Thanks for your good suggestion. We evaluate commonly employed forecasting models, analyzing their respective application scenarios and distinguishing characteristics, as summarized in Table 3. Through this comparative analysis, Model Predictive Control (MPC) emerges as the optimal approach for our research framework. We added Table 3 and the following sentence to paragraph 1 of the 4.1 section:

“To address the multi-time scale scheduling challenges in IES, numerous forecasting methods and optimization techniques - extending beyond Model Predictive Control (MPC) - can be utilized to improve scheduling performance. Table 3 presents a comparative analysis of prevalent forecasting approaches along with their distinctive application characteristics. Based on the comparative evaluation of prediction model attributes presented in the table, MPC has been selected as the most suitable approach for this study.”

Table 3 Comparison of Prediction Methods in Multi-Timescale Scheduling

| **Comparison of Application Scenarios** | | | |
| --- | --- | --- | --- |
| **Forecasting methodology** | **Applicable time scale** | **Advantages** | **Limitations** |
| MPC (Model Predictive Control) | Real-time / Intra-day | Rolling optimization, strong constraint handling | High computational complexity |
| LSTM (Long Short-Term Memory) | Day-ahead | Captures long-term dependencies | Requires large training datasets |
| ARIMA (Autoregressive Integrated Moving Average) | Short-Term / Medium-Term | Simple, highly interpretable | Poor adaptability to nonlinear data |
| DRL | Joint Prediction-Decision Optimization | Enables end-to-end optimization | Unstable training, hyperparameter sensitivity |

5. It is recommended to include a brief discussion on future work in the conclusion to provide a forward-looking perspective.

**Response**: Thanks for your good suggestion. We have modified the manuscript and added the following sentence to the 6-th section:

“To further advance this field, future research could prioritize the following directions:

(1) Computational Efficiency for High-Dimensional Systems – Future studies should focus on developing lightweight MPC variants to enable real-time control in large-scale systems, including distributed energy networks and multi-agent cooperative systems. Further exploration of edge computing integration could enhance computational scalability.

(2) Robustness Under Uncertainty – Enhancing the current framework to address model mismatch, non-Gaussian disturbances, and adversarial scenarios—potentially through distributionally robust optimization or adaptive learning techniques—would significantly improve practical applicability in uncertain environments.

(3) Dynamic Granularity Adaptation – The development of self-adaptive time-scale partitioning algorithms, such as those leveraging event-triggered mechanisms or digital twin feedback, could dynamically optimize scheduling granularity in response to renewable forecasting errors and demand fluctuations.

(4) Equipment Dynamics and Robustness Verification – Future investigations should systematically examine the dynamic interactions between equipment response characteristics and system-level stability, such as comprehensive parameter sensitivity analysis of device response rates and development of integrated robustness testing frameworks capable of evaluating the coupled dynamics-stability relationships in multi-energy scenarios.

These research directions aim to bridge current theoretical and practical gaps while enhancing the applicability of multi-timescale optimal scheduling methodologies for integrated energy systems in complex real-world scenarios.”

**Reviewer #2:**

Thank you for taking time out of your busy schedule to read our research work again. Your valuable suggestions are very helpful for the revision of our paper. We have read your opinions carefully and revised our manuscript. We hope this revision can meet your standards. Here are the point-to-point responses.

1. The CNN-ATT-BiLSTM model proposed in the paper is only compared with CNN and BiLSTM, and other mainstream prediction models (such as Transformer, GRU, etc.) or hybrid models (such as CNN-LSTM) are not included as baselines, making it difficult to comprehensively demonstrate its superiority.

**Response**: We are very grateful for your suggestion. We have increased the comparison with the hybrid model CNN-LSTM and modify Figure 3-4 and Table 2 in 3.3 section. We also modified the manuscript and added the following sentence to last paragraph of 3.3 section:

**Fig 3 The Predicted Waveforms of WT Power**

**Fig 4 The Predicted Waveforms of PV Power**

**Table 2 Prediction Errors of Different Models**

|  | **Prediction model** | **RMSE(kW)** | **MAE(kW)** | **R^2^/%** |
| --- | --- | --- | --- | --- |
| **WT power** | CNN | 24.314 | 16.538 | 0.892 |
|  | BiLSTM | 18.424 | 11.436 | 0.924 |
|  | CNN-LSTM | 13.825 | 7.29 | 0.968 |
|  | CNN-ATT-BiLSTM | 11.937 | 6.127 | 0.981 |
| **PV power** | CNN | 12.314 | 7.538 | 0.902 |
|  | BiLSTM | 9.424 | 6.936 | 0.931 |
|  | CNN-LSTM | 7.902 | 5.834 | 0.976 |
|  | CNN-ATT-BiLSTM | 7.137 | 5.127 | 0.987 |

“The comparative analysis of prediction performance, as illustrated in Figures 3-4 and Table 2, demonstrates that CNN-LSTM achieves superior performance to the standalone CNN and BiLSTM model, exhibiting reduced prediction error and enhanced accuracy. However, the proposed CNN-ATT-BiLSTM architecture emerges as the most effective model, with the minimal prediction error and an R2 value approaching unity, indicating optimal predictive capability.

This advanced hybrid model integrates three complementary mechanisms: CNN for extracting local spatial features, BiLSTM networks for capturing comprehensive temporal dependencies in both forward and backward directions, and ATT for dynamically emphasizing critical temporal features. The CNN-LSTM architecture, while maintaining spatial feature extraction capabilities through its CNN component and simplified structure, demonstrates effective performance for stationary time series but fails to account for reverse temporal dependencies. The standalone CNN model shows proficiency in identifying local patterns and periodic fluctuations, yet exhibits limitations in modeling long-term temporal relationships. The BiLSTM network, although capable of bidirectional temporal context learning, lacks spatial feature extraction capacity.

These comparative results clearly demonstrate the superior modeling capability of the CNN-ATT-BiLSTM architecture, which effectively combines the strengths of spatial feature extraction, bidirectional temporal modeling, and attention-based feature weighting. The model's exceptional performance is particularly evident in renewable energy power prediction applications, where it successfully captures both spatial correlations and complex temporal dynamics simultaneously.”

2. The data only mentions "Data will be made available on request", but the specific sources, time range, or preprocessing methods are not clearly stated.

**Response**: Thanks for your good suggestion. We have modified the manuscript and added the following sentence to paragraph 1 of the 5-th section:

“This study employs operational dataset from an industrial park in Jiangsu Province, China, covering the period from May to July over a 24-year span, with the original data being provided and validated by Jiangsu Pinggao Tai Shida Electric Co. LTD under a confidentiality agreement. The simulation is conducted in MATLAB R2023a and solved by Gurobi solver, with detailed equipment parameters provided in Table 4.”

3. The scenario comparison only shows the results of the total cost and carbon emissions, lacking a quantitative analysis of the specific scheduling strategies (such as the start-stop timing of equipment and the charging and discharging modes of energy storage). It is recommended to supplement dynamic diagrams of the scheduling process during typical periods (such as the peak and valley electricity price periods) and explain how DR and the carbon trading mechanism collaborate to optimize the output of equipment.

**Response**: We are very grateful for your suggestion. We increase the peak and valley load scheduling under demand response. We also added Fig 6, Fig 7 and following sentence to 4.2 section:

**Fig 6 PDR and IDR Power at different time scales**

**Fig 7 The Waveforms of PDR and IDR Power**

“Fig 6 presents the power dispatch diagrams for day-ahead PDR, day-ahead IDRA, intra-day IDRB, and real-time IDRC. The red segments indicate shiftable loads during the current period, while the green segments represent curtailable loads. Owing to fluctuations in time-of-use electricity pricing, load demand, and renewable energy generation, the demand-responsive loads consistently shift from peak-price periods to valley-price and shoulder-price periods. Fig 7 compares the PDR power profile with the aggregated power profiles of the three IDR types. From these results, the baseline power load profile - both with and without demand response - can be derived, as illustrated in Fig 8. This approach effectively achieves peak shaving and valley filling while facilitating renewable energy integration and stabilizing power fluctuations.”

4. The literature review part does not fully cover relevant studies in recent years (such as multi-time-scale scheduling based on reinforcement learning, integration of hydrogen energy storage, etc.), and there is a lack of critical analysis of the disadvantages of existing methods. It is recommended to expand the scope of the literature and clarify the unique contributions of the model in this paper compared with similar works.

**Response**: We are very grateful for your suggestion. We have modified the manuscript and added relevant literature.

“The dynamic dispatching process of demand response (DR) represents a closed-loop control mechanism that enables power systems to dynamically optimize demand-side resources - including flexible loads, energy storage systems, and electric vehicles - in response to real-time grid conditions, pricing signals, or dispatch commands. This sophisticated process achieves multiple objectives: peak shaving, supply-demand balancing, and enhanced system flexibility and economic efficiency. Ref. [13] proposes a tri-level DR framework to enhance the flexibility of electric vehicle charging stations (EVCS) in coupled power-transportation networks (CPTN) by integrating distribution network operators (DNOs). The framework employs deep reinforcement learning (DRL) to dynamically balance profit maximization and operational safety, the results demonstrate superior EVCS operational flexibility compared to traditional charging load regulation methods. Ref. [14] integrates micro-urban transportation network (MUTN) with distribution network (PDN) to realize high-precision vehicle-to-grid (V2G) analysis. It combines SUMO for MUTN simulation and optimized DistFlow model for PDN analysis, and combines the detailed charging dynamics of fast charging station (FCS) and slow charging station (SCS). Ref. [15] proposes a resilient hierarchical load frequency control (LFC) strategy for microgrids (MGs) integrating distributed renewable energy sources (DRESs), particularly addressing wind power fluctuations. Ref. [16] proposes a novel distributed hybrid-triggered (HTed) secondary control (SC) scheme for DC microgrids (MGs) that combines event-triggered communication with self-triggered sampling/computation to optimize efficiency.”

5. The paper assumes that the response rates of all equipment are fixed (such as the ramp rate of the Micro-Turbine (MT) and the charging and discharging efficiency of energy storage), but does not discuss parameter sensitivity or conduct robustness tests (such as the system stability when the prediction errors of wind and solar power increase).

**Response**: We appreciate this insightful observation. The current study indeed adopts fixed efficiency and ramp rates for modeling simplicity, as our primary focus was to establish a baseline framework for intra-day scheduling, coupled with seamless integration into real-time optimization strategies, thereby enhancing operational efficiency and decision-making precision. We introduced slack variables to ensure feasible dispatch across stages and added the following sentence to last paragraph of 4.1 section:

“Additionally, energy storage systems are leveraged to further compensate for forecasting errors and enhance operational flexibility. In the intra-day adjustment layer, MT2 is designated as the slack variable to absorb power imbalances, whereas in the real-time control layer, FC serves as the slack variable to ensure instantaneous power balance.”

We fully acknowledge that parameter sensitivity and prediction error robustness warrant further investigation. These factors will be prioritized in our future work, particularly for real-time control scenarios where dynamic responses are critical. We have modified the manuscript and added the following sentence to the 6-th section:

“To further advance this field, future research could prioritize the following directions:

(1) Computational Efficiency for High-Dimensional Systems – Future studies should focus on developing lightweight MPC variants to enable real-time control in large-scale systems, including distributed energy networks and multi-agent cooperative systems. Further exploration of edge computing integration could enhance computational scalability.

(2) Robustness Under Uncertainty – Enhancing the current framework to address model mismatch, non-Gaussian disturbances, and adversarial scenarios—potentially through distributionally robust optimization or adaptive learning techniques—would significantly improve practical applicability in uncertain environments.

(3) Dynamic Granularity Adaptation – The development of self-adaptive time-scale partitioning algorithms, such as those leveraging event-triggered mechanisms or digital twin feedback, could dynamically optimize scheduling granularity in response to renewable forecasting errors and demand fluctuations.

(4) Equipment Dynamics and Robustness Verification – Future investigations should systematically examine the dynamic interactions between equipment response characteristics and system-level stability, such as comprehensive parameter sensitivity analysis of device response rates and development of integrated robustness testing frameworks capable of evaluating the coupled dynamics-stability relationships in multi-energy scenarios.

These research directions aim to bridge current theoretical and practical gaps while enhancing the applicability of multi-timescale optimal scheduling methodologies for integrated energy systems in complex real-world scenarios.”

6. It is necessary to unify the format of the charts, supplement the necessary annotations, and check the continuity of the formula numbering.

**Response**: Thanks for your good suggestion. We added some charts, reprocessed the previously unclear graphs, and rechecked the formula numbering. After a comprehensive inspection, we confirm that all the specified elements meet the required standards and are correct. After comprehensive examination, we confirm that all specified elements meet the required standards and are correct as presented.

Once again, thank you for taking the time to review our work. Your constructive comments and suggestions would help us to develop research work and improve the quality of the paper in depth.

Best regards

Yours sincerely

Dengfeng Zhang

Institute of Intelligent Manufacturing

Nanjing Tech University

Jiangsu Province, China, 211816

Email: zhdfnjtech@163.com
